# Migration and the Multi-Dimensional Well-Being of Elderly Persons in Georgia

**DOI:** 10.1007/s12062-017-9176-4

**Published:** 2017-02-12

**Authors:** Jennifer Waidler, Michaella Vanore, Franziska Gassmann, Melissa Siegel

**Affiliations:** Maastricht Graduate School of Governance, UNU-MERIT, Boschstraat 24, 6211 AX Maastricht, The Netherlands

**Keywords:** Elderly, Georgia, Migration, Multi-dimensional well-being

## Abstract

High rates of migration coupled with low formal social protection provisions may place many members of the elderly Georgian population in precarious living conditions that promote vulnerability and limit well-being achievement. This potential connection has been poorly explored in past literature, however, suggesting a need to better assess how the migration of an adult child may influence the multidimensional well-being of the elderly in Georgia. Using a novel dataset comprising 2202 elderly individuals across all regions of Georgia (excepting the territories of Abkhazia and South Ossetia), this paper proposes a multidimensional well-being index that has been specifically designed to encompass the unique resources and constraints faced by elderly individuals in different age cohorts. Following the construction of a multidimensional well-being index—comprised of domains including physical health and independence, housing well-being, social well-being, and emotional well-being—the outcomes of elderly individuals are compared by age and the presence/absence of adult children due to migration. Findings suggest that the migration status of an elderly person’s adult children is related to the attainment of well-being. Elderly individuals with a migrant child are more likely to attain well-being in physical health as well as in the overall multidimensional well-being index.

## Introduction

Amidst economic and political transition, civil conflict, and precarious living conditions, international migration has become an attractive livelihood strategy for many Georgians over the past two decades. The risks of migration for the vitality of the family have been documented at length, yet limited focus has been paid to the potential risks or benefits of the migration of adult children for the well-being of their elderly parents “left behind”. Scant information is available on the situation of elderly persons in Georgia in particular, yet the rapid growth of the elderly population, imminent demographic crisis, and ongoing economic challenges have highlighted the need to better understand how mobility and elderly well-being may be interrelated.

Since the dissolution of the Soviet Union in 1991, Georgia has experienced significant emigration flows. As of 2010, 25.1% of the total population was estimated to live abroad, concentrated in the Russian Federation, Armenia, Ukraine, Greece, and Israel. The majority of recent migrants are working-age adults seeking better employment opportunities abroad. The labour-driven nature of Georgian migration has brought with it sizeable remittance transfers, which accounted for US $824 million in 2010 (World Bank 2010). Remittances can play a powerful role in reducing poverty, particularly as a significant number of families rely on remittances as the only source of income (Gassmann et al. 2013). Migration is not an exclusively economic phenomenon, however, and the migration of adult children can potentially deprive older individuals of vital sources of care. As of 2010, it was estimated that nearly 60% of all migrants were female (ibid.), a phenomenon that could contribute to potential withdrawal of care for elderly kin. Many migrant women are destined for the elder-care sector abroad, which has grown in many European countries and created new economic niches for large numbers of migrant women (Cangiano and Shutes 2010; Anderson 2012).

In recognizing that migrants are not only potential income earners but also potential providers of care and other resources, it becomes key to understand if and how the well-being of the elderly as a unique population group may be influenced by the absence of a migrant child. This paper investigates the potential implications of adult child migration for the well-being of their elderly kin who remain in Georgia by proposing an elderly-specific multidimensional well-being index that compares elderly well-being outcomes according to child residency status. Before explaining the creation of the multidimensional index, however, the first section describes the intersection between migration and elderly well-being. Following this, well-being itself is described and defined, and the data and methodology followed is then explained. The next section describes the index and its results, and the paper then concludes with a discussion of results.

## Migration & Elderly well-Being

In much development and migration literature, migration is discussed as a catalyst for development and a supplement for weak or ineffectual state structures. Several mechanisms—namely the transfer of monetary and social remittances (UNDP 2009)—have received particular focus as means of reducing vulnerabilities among the members of a migrant’s family. Much literature has emphasized that migration can bear both positive and negative consequences on the well-being of the “left behind”, however, particularly for specific population groups such as the elderly.

The (new) economics of labour migration (NELM) theory has suggested that migration is a household-level decision undertaken in response to market failures (such as missing or imperfect credit and insurance markets) that leave households vulnerable to economic shocks. Migration is envisioned as a way to expand not only the volume of income but to diversify income sources, which can protect the household against particular types of shocks while also enabling the accumulation of income for expenditures on future income-generating activities (Stark and Bloom 1985; Taylor 1999; Massey et al. 1993). This theory would suggest that the decision to migrate is made collectively on the household level, and elderly persons would be expected to experience some increases in well-being, particularly in external resources such as material living standards. In the absence of formal state social protection provisions and adequate employment, as is the case in Georgia, migration could indeed present a valuable household livelihood strategy. A migrant is not only a potential source of income, however, but also a household and family member who fulfils a diversity of roles. One of those roles may be as a caregiver, which is particularly important in societies like Georgia where social protection measures, including formal elderly care, do not adequately meet the needs of the ageing population.

Many states in the former Soviet Union, including Georgia, have undergone intense economic and demographic transitions. In Georgia this transition is expected to result in a 15.7% increase in the population aged 60 and above between 2012 and 2050, with the 60+ population group representing over 35% of the total population by 2050 (UNFPA 2012). The World Bank has predicted that between 2000 and 2025, the total population will decrease by 17% (Chawla et al. 2007); while this reflects a shortfall of new births, migration will likely contribute to this change (IOM 2003). The growth in the elderly population coupled with population loss is expected to significantly increase the old-age dependency ratio (Chawla et al. 2007). What is notable in the Georgian context, however, is the high rate of participation of elderly persons in the workforce. A significant portion of the population remains economically active well into retirement age, with 21% of the labour force represented by individuals aged 60 or older. While increasing life expectancy is one factor that contributes to this pattern, inadequate social protection provisions also contribute to the need for the elderly to keep working (World Bank 2009).

The demographic transition and economic vulnerability of the ageing population has important implications for elderly care. Social protection measures for the elderly are unlikely to improve given the increase in the old-age dependency ratio, likely increasing the economic burden faced by families caring for their elderly kin. These challenges are compounded by fundamental changes to the labour market that have resulted in more working-age adults moving to urban areas in search of work, leading to changes in family residency patterns and elderly care practices (King and Vullnetari 2008). Migration beyond state borders in particular can weaken informal social care systems that are poorly supplemented by formal support systems (Grant et al. 2009). The absence of adult children as well as other young members of extended social networks can contribute to a care drain in which elderly individuals face the ageing process without access to the (physical) external resources traditionally provided by kin networks.

Despite increased attention to the potentially dramatic implications of migration and demographic changes on elderly care and subsequent well-being, studies on intergenerational relations, mobility, and ageing suggests that changes in family structures, including through migration, can have differing consequences for elderly persons. For example, several studies conducted in Mexico have linked the emigration of adult children to deteriorating physical health of elderly individuals, particularly among those who lack physical support in routine daily activities following a post-migration transition to independent living arrangements (Kanaiaupuni 2000). Antman (2010)), also in Mexico, found that elderly persons with adult children living abroad were more likely to experience a heart attack or stroke as well as higher levels of self-reported health deterioration. Both the Kanaiaupuni and Antman studies further suggested that elderly parents of migrants were more likely to experience deteriorating emotional health, which could potentially compound declining physical health.

Other studies in Eastern Europe have suggested that the migration of adult children can contribute to poor emotional health outcomes among the elderly, particularly following rapid structural and socio-economic changes that have led to changes in family care and residency patterns. In both Moldova (Grant et al. 2009) and Albania (King and Vullnetari 2006), elderly persons have reported feelings of loss and abandonment following the migration of adult children. The particular post-migration changes to elderly well-being are largely context dependent, however. In Thailand a study by Abas et al. (2009) found that the elderly parents of children in migration reported lower levels of depression, which could potentially reflect positive repercussions of remittance receipt as well as the migrant selection process (in which individuals from relatively wealthier and better-educated households, whose members are less likely to be depressed because of the better socio-economic status, are more likely to enter migration).

Changes in both physical and emotional health may reflect other adjustments made to the daily activities brought about by migration. Where Kanaiaupuni (2000) found a link between residency patterns and declining well-being, other studies have found that changes to domestic tasks necessitated by migration may also affect elderly well-being. In China, Chang et al. (2011) found that elderly individuals in households containing a migrant spent more time on domestic tasks and both farm and off-farm work, with women experiencing the greatest increase in time spent working. Shifts in domestic caring tasks may present increased sources of stress to elderly individuals. When adult children migrate, elderly individuals may assume the primary caregiving responsibilities for grandchildren, which some individuals may not have the resources or capabilities for (Salah 2008; Prohnitchi 2005), particularly those with limited financial means and limited access to state child-care benefits (HelpAge International 2008). Increased child care tasks need not translate into more limited resources, however. As highlighted by Cong and Silverstein (2012) in their study of intergenerational support patterns in China, older persons who care for their grandchildren may receive more emotional and financial support from their adult children. This study highlights that elderly persons should be understood as members of wider family networks that can mobilise resources and support in response to different types of changes in family circumstances, including migration.

Results from past studies largely confirm what theory would lead one to expect: well-being is comprised of mutually-reinforcing domains that can each be affected by migration in different ways. Anticipating the direction and magnitude of these changes requires appropriate understanding of country and culture-specific contexts, as both formal social protection provisions and informal caregiving expectations can result in different levels of elderly vulnerability.

## Defining well-Being

The first step in assessing the potential impacts of migration on the well-being of elderly individuals requires knowing *what* well-being is and how it should be measured. Well-being can be understood both as a theoretical construct—formulated to encompass rights-based approaches to development—and as a functional concept—formulated for ease of measurement and comparison.

From a theoretical perspective, the capabilities approach provides valuable conceptual insight into the components of well-being. The capabilities approach is rooted in the notion that well-being is multidimensional and is the product of an individual’s effective opportunities to do or become that which he or she so desires (“functionings”). An individual’s opportunities, or capabilities, determine the functionings that an individual can achieve; lack of capabilities, or the freedom to choose among them, leads to limited realizable functionings—deprivation or poverty (Sen 1993; Robeyns 2005). Functionings span multiple dimensions of an individual’s life that contribute to each person’s sense of worth and fulfilment. Deprivation in any number of dimensions can thus result in the failure of an individual to achieve well-being (Alkire 2002; Sen 1993; Robeyns 2005; Alkire and Foster 2011).

While within this theoretical framework there is some controversy over what “universal”, key functionings constitute well-being, defining the components of well-being is necessary for well-being to be measured. Given changes to capabilities and resources over time, components of well-being differ across different life stages. In recognising this, the World Health Organisation proposed that quality of life is: an “individual’s perception of their position in life in the context of the culture and value systems in which they live and in relation to their goals, expectations, standards and concerns” (WHOQOL 1995, p.1405). This definition was formulated in 1995, when the WHO launched an initiative to develop an international assessment of quality of life that would function across cultures and generations. Within this initiative a tool was developed to provide a multidimensional profile of quality of life scores clustered around six domains and 24 sub-domains. Those domains—physical health, psychological health, level of independence, social relationships, environment, and personal beliefs—are intended to represent universal concerns that can be tailored to different populations according to the included subdomains (WHOQOL 1995).

The one-size-fits-all quality of life measure proposed by WHO provides important initial cues about the components of elderly well-being, but many tools have been developed explicitly for the ageing population. Elderly well-being can be usefully understood as an outcome of successful ageing and adaptation (Brandtstädter and Greve 1994). Different authors working from perspectives such as gerontology, healthcare, and psychology have suggested that successful ageing is reflected in different domains of wellness. A number of multilevel assessment instruments have been designed to measure elderly well-being and quality of life, most of which contain both subjective and objective indicators of well-ness. Table 1 below summarises various definitions of quality of life and the components of well-being they encompass.

**Table 1:** Summary of Elderly Well-Being Instruments

Author/ Institution	Instrument	Domains
George and Bearon (1980) as cited in Farquhar (1995)		General health & functional status
		Socioeconomic status
		Life satisfaction
		Self-esteem
Lawton et al. (1982);1983) as summarised in Brown et al. (2004)		Behavioural competence (cognitive dimensions of health and social behaviour)
		Perceived quality of life
		Psychological well-being
		Objective environment (such as material living conditions)
Lawton (1975)	Philadelphia Geriatric Centre Assessment	Physical health
		Social interaction
		Time use
		Cognitive (including mental health) functioning
		Activities of daily living
		Perceived environment (housing conditions, access to community services community, etc.)
		Personal adjustment (including indicators of emotional wellbeing)
Farquhar (1995)		Health and mobility
		Family relationships
		Social contacts
		Activities
		Emotional well-being
		Material circumstances
Cummins (2003)		Relationships
		Activities
		Health
		Life philosophy
		Personal life history
		Meaningful future outlook
Lau et al. (2003)	Comprehensive Quality of Life (ComQol)	Health-related elements (as pain, mobility, & activities of daily living)
		Subjective well-being (life satisfaction or happiness)
		Social factors (interpersonal relationships & social support)
Fillenbaum (1984)		Activities of daily living
		Mobility
		Mental Health
		Physical health
		Social & economic functioning
Coughlin (2010)	Gallup-Healthways Well-being Index	Life evaluation
		Emotional health
		Physical health
		Healthy behaviour
		Work environment
		Basic access
Kaneda et al. (2011)	Standford Center on Longevity index	Emotional well-being
		Social well-being
		Material well-being
		Physical well-being (including mobility & nutrition)

A number of elderly well-being studies from different fields such as gerontology and psychology have elaborated functional definitions of quality of life that contain common elements. All of the studies and instruments summarised envision well-being as inherently multidimensional and as a product of functional opportunities complemented by achieved outcomes. All have identified physical health and mobility, emotional health and social interaction, and material living standards (including the environment) as key components of well-being. As suggested by Cummins (1996; 1999), almost all measurement methods also reflect the notion that well-being is comprised of both culturally-relevant objective dimensions and respondent-weighted subjective dimensions.

The summarised studies synchronise well with the capabilities approach, as they explicitly recognise age-specific characteristics and constraints that challenge the realisation of opportunities into functionings. As an individual ages, the relationship among functionings grows closer; changes in one domain of well-being often necessitate changes to the others. As an example, the deterioration of basic mobility and physical independence, which has implications for an individual’s ability to maintain social relationships, can reinforce deteriorating emotional wellness (Fillenbaum 1984; Ward et al. 2012). Deprivation in any given dimension will thus likely contribute to deprivation in another, which not only compounds the incidence of deprivation but the intensity of deprivation as well (Ward et al. 2012). A further aspect of measuring well-being of the elderly is that the relative weight given to different functionings is likely to change as a person grows older: while individuals in the “young old” age cohort were found to value material living standards the most, the oldest members of the elderly cohort were most concerned with mobility and lack of aid (Farquhar 1995). The mutually-reinforcing nature of well-being domains as well as the shift in domain value over age requires that any method for measuring well-being is sensitive to heterogeneity within the group. While “the elderly” are referred to as one (homogenous) population, age is an imperative differentiating factor: throughout analysis of well-being, age must be taken into account when defining thresholds for “normality”.

In drawing from the diversity of definitions of well-being and quality of life posed by past research, and in understanding challenges to their measure, the following definition of well-being will be used for this study:

Well-being is a multidimensional state of personal being comprised of both self-assessed (subjective) and externally-assessed (objective) positive outcomes across five realms of opportunity: physical health or well-being, emotional health, housing standards, and social well-being.

This definition brings together the common well-being domains suggested by past studies, and in doing so it recognises that well-being is a product of a multitude of opportunities within an individual’s life. These elements are seldom context independent and static, changing not only with age but as the result of other complex processes. Migration is one such process that alters the context in which individuals function, but its potential consequences are not universal and homogenous.

## Data & Methodology

### Data

Given the scale of the emigration phenomenon from Georgia coupled with the growing share of elderly within the total population, Georgia provides an excellent case study through which the relationship between migration and the well-being of the elderly “left behind” can be explored. Analysing migration-related trends in Georgia has been a problem in the past due to limited data on migration and a lack of nationally-representative data. This analysis benefits from data collected via a nationally-representative household survey implemented within the European Commission-financed study “the Effects of Migration on Children and the Elderly Left Behind in Moldova and Georgia”. The survey was explicitly designed to explore the possible consequences of familial separation through migration on the well-being of children and the elderly. In the absence of a nationally-representative sampling frame, one was elaborated on the basis of recently-updated election registration lists. Households were then selected via the random route method, and the survey was implemented among those households that contained one or more children or elderly persons. The survey collected information on the demographic features of household members, household living conditions, members’ migration histories, and the experiences and conditions of elderly household members. Migrants were identified as “any individual who lived abroad for three or more months consecutively at the time of the survey”. To retain elderly individuals as the unit of analysis, information was collected directly from individuals over the age 60 about work history, time allocation, physical health and nutrition, mental health, mobility, and relationships with household and non-household members*.*

Data was collected among 4010 households across all regions of Georgia between March and December 2012,^1^ nearly two-thirds of which contained one or more individuals aged 60 or above. The total survey sample included 3407 elderly individuals, 2202 of which were included in the final analytical sample. As this analysis is concerned with differences in well-being outcomes of individuals with and without adult migrant children, individuals *without* adult children were excluded from the sample. Only those individuals who provided information on all indicators used in the analysis were retained for the final sample as well, which reduced the sample size to 2202 observations.

Key characteristics of the analytical sample are provided in Table 2. As can be seen from the table, a slightly larger proportion of elderly persons belong to the older age cohort, and women far outnumber men. In terms of household composition, almost half of elderly individuals live with other adults, followed by more than 35% of the elderly sample who live in households with at least one child below 18 years old, indicating a high incidence of multigenerational households. Approximately 10% of older individuals live with their partner, and the remaining 7.8% live alone.

**Table 2:** Key Characteristics of Elderly Sample

Age Cohort	60–69 years		70 and older		Total	
	# obs	%	# obs	%	# obs	%
Gender						
Male	386	38.0	415	35.0	801	36.4
Female	631	62.0	770	65.0	1401	63.6
Residency Arrangement						
Alone	46	4.5	125	10.6	171	7.8
With partner	72	7.1	144	12.2	216	9.8
With other adults	473	46.5	539	45.5	1012	46.0
With children	426	41.9	377	31.8	803	36.5
Migration status of children						
Migrant child	412	40.5	422	35.6	834	37.9
No migrant child	605	59.5	763	64.4	1368	62.1
Region						
Rural	425	41.8	611	51.6	1036	47.1
Urban	592	58.2	574	48.4	1166	53.0
Total	1017	46.2	1185	53.8	2202	100

*Source: Author’s calculations*

Given the focus of this analysis on the connections between migration and elderly well-being, the sample of elderly individuals was split in two groups according to the migration status of the elderly person’s adult children. According to the sample, almost 38% of elderly individuals have at least one child living outside Georgia; the proportion is slightly higher for the youngest cohort (40.5% against 35.6%). Finally, a slightly larger percent of the elderly population live in an urban area, although this result is driven by the youngest cohort as a slightly higher number of elderly above 70 years old live in rural areas.

### Indicators

The focus of this multidimensional well-being analysis necessitates the construction of an elderly-specific index comprised of indicators representing possible attainments within four domains of well-being. In line with the definition of well-being provided above, such an index was chosen for its multidimensional structure, inherently comparative nature, and replicability. While such an index allows for identification of the proportion of the population that can be considered well or deprived, it more importantly allows for comparison of well-being attainment across population groups and per dimension and indicator.

The present elderly well-being index (EWB) contains four dimensions: physical, social, emotional, and housing well-being. Within this index, indicators of well-being were selected based on their appropriateness in capturing well-being in the Georgian context; indeed, single-country studies advantageously allow for the selection of indicators and thresholds to be tailored to local norms and values (Roelen et al. 2009). The indicators also reflect the possibilities and constraints of the survey data. Most indicators retained the elderly individual as the unit of analysis, as information on opinions and achievements was collected from elderly respondents directly. Indicators reflecting material living conditions, however, reflect the situation of the entire household; each individual within the household is assumed to experience the same conditions. Table 3 summarises the indicators selected per domain to represent the well-being of elderly individuals.

**Table 3:** Well-being Indicators per Dimension

Dimension	Indicator(S)
Physical well-being & independence	• Individual has retained essential mobility functions • Individual does not have difficulty self-administering medications
Housing well-being	• Individual is living in a house with appropriate flooring, electricity, and access to safe water
Social well-being	• Individual has regular contact with family or friends
Emotional well-being	• The individual is satisfied with his/her current life • The individual is not depressed

Physical well-being is comprised of two indicators. The first measures the elderly individual’s ability to perform activities of daily living (basic mobility functions) such as bathing, dressing, walking, and going to the bathroom without assistance. The mobility indicator is a composite measure created through factor analysis, which was conducted to determine the underlying factors that explain rates of mobility. For the factor analysis, we created several binary variables measuring elderly individual’s ability to perform essential daily functions, all of which were correlated with each other. The second physical well-being indicator measures an individual’s ability to take medication without aid, which is used as a proxy of functional independence. The ability to self-administer is correlated with other activities measuring independence given the levels of mental cognisance required (Kaneda et al. 2011).

Housing well-being is the domain that captures material living standards. Elderly individuals who live in homes with appropriate flooring (e.g., not unpolished wood, dirt, or clay), with electricity, and with access to safe drinking water (e.g., not from surface water or rainwater collection) are considered well-off in this dimension. Housing conditions rather than more traditional indicators of material well-being such as income or expenditures were included for two reasons. The first is that reporting of income/expenditure data is often unreliable, and the second is that incomes/expenditures are likely to shape the attainment of well-being in other domains and should thus be included as a control in multivariate analyses.

The dimension of social well-being encompasses relationships with family and community members, as both types of social ties are important in shaping well-being outcomes. Extensive literature supports the idea that a good relationship with family and people in the community helps improve overall elderly well-being (Ward et al. 2012; Kaneda et al. 2011; Fillenbaum 1984). Care support from family and friends–or the lack thereof as a consequence of living far away from each other—has been identified as an important component of social functioning.

While many instruments exist for measuring the dimension of emotional well-being, there is limited consensus on the best tool to use, on standards of measurement, and on thresholds for defining deprivation or health, particularly across disciplines. Based on previous studies and on the available data, the indicators chosen to measure emotional health were self-reported depression and self-reported current life satisfaction. These two indicators indicate level of self-perceived wellness. Depression and life satisfaction were measured using a set of questions designed for the mental health inventory (MHI-38), an instrument designed to measure mental health among the elderly (Department of Health and Ageing 2003). The choice to measure depression using self-reported questions reflects the view that self-reported measures are usually better than clinical diagnostic tools, as they measure causes of late-life depression, such as coping with chronic illnesses, disability, feeling of loneliness, etc. (Kaneda et al. 2011). The indicator of life satisfaction was measured using a ten-point Likert scale in which respondents rated satisfaction with their current life. Based on the Cantril Self-Anchoring Striving Scale,^2^ a score of seven or higher indicates that an individual is “thriving” or satisfied with his/her own life.

### Methodology

The purpose of the empirical analysis is to assess elderly well-being and compare the well-being of elderly persons with and without a migrant child. A step-wise approach was adopted for the analysis. First, well-being with respect to each indicator was analysed separately. An elderly individual can be considered not deprived if s/he meets the established well-being threshold set for a given indicator. Indicator well-being rates *(IWB)* are calculated by counting the number of elderly persons who meet the requirement and are expressed as a share of all the elderly (Roelen et al. 2011; Roelen and Gassmann 2012)^3^:

$$ { I WB}_x=\frac{1}{n}\sum_{i=1}^n{I}_{i x} $$

where n is the number of elderly for which the indicator is observable and *I*_*ix*_ is a binary variable taking the value 1 if the elderly person *i* has reached the threshold and 0 if the elderly person has not with respect to indicator *x*. The denominator, *n*, differs across indicators depending on the number of actual observations. Indicators observed at household level, such as for monetary well-being or housing, are translated to all elderly persons living in the respective household, assuming equal access and intra-household distribution.

A second step involved building a multidimensional well-being index inspired by the methodology developed by Alkire and Foster (2011) for the measurement of multidimensional poverty. This is a well-established methodology in the field of poverty and wellbeing analysis and it underlies the global multidimensional poverty index annually published in the Human Development Report since 2010 by UNDP. An elderly person is considered to be multidimensionally well if the weighted combination of indicators is equal to or exceeds 70% of the total. The decision to set the cut-off at 70% of the aggregated indicators follows the cut-off used for multidimensional child well-being indices (e.g. Roelen and Gassmann 2012) and reflects the opposite of the 30% threshold used when measuring levels of deprivation (see, e.g. Alkire and Foster 2011). Each domain is assigned equal weight and each indicator within a domain is also equally weighted (see Table 4 below). This facilitates the interpretation of results (Atkinson 2003) but also asserts that each dimension is considered of equal importance. Weights can be determined in various ways, such as through participatory processes, based on expert opinion, or derived from survey data. Calibration of weights depends on the information available; in the absence of information on the relative value or importance of specific dimensions (or indicators), equal weights are chosen. The lower the well-being cut-off, the higher is the share of elderly doing well and the lower is the average intensity of well-being.

**Table 4:** Indicator Well-being Rates: Dimensions & Weights

Dimension	Indicator	Weights in IWB
Physical well-being	Individual has retained basic mobility functions	1/8
	Individual has no difficulty self-administering medication	1/8
Housing well-being	Individual is living in house with appropriate floor, electricity and access to safe water	1/4
Social well-being	Individual has regular contact with family or friends	1/4
Emotional well-being	Individual is not depressed	1/8
	Individual is satisfies with current life	1/8
Total IWB		1

In establishing the multidimensional well-being index, all elderly individuals who are well in any indicator are identified and subsequently assigned the indicator weight, or zero if they have failed to attain wellness. An elderly person is considered well if the sum of the weighted indicators is equal to or higher than the cut-off value. Elderly individuals with positive outcomes are then assigned a value of one; all others are assigned a value of zero. The incidence (or headcount rate) of multidimensional well-being is the percentage of elderly individuals considered well as a proportion of all elderly individuals.

The following section describes the results of the multidimensional index. Descriptive statistics for indicator and multidimensional well-being are presented, and multivariate analysis is subsequently applied in order to test for group differences and to identify other correlates that determine elderly well-being, such as personal characteristics of the elderly person and household characteristics. Separate binary outcome models are estimated for selected indicators using standard probit models:

$$ \Pr \left({y}_i=1|{x}_i\right)=\Phi \left({x}_i\beta \right),\kern0.5em \mathrm{with}\ \mathrm{i}=1,\dots, \mathrm{N} $$

Where *y*_*i*_ is the binary outcome variable, *Φ* is the standard normal distribution function, ***x***_***i***_ is a vector of explanatory variables, and ***β*** is a vector of coefficients to be estimated. In this case the dependent variable is the probability that an individual is vulnerable with respect to a specific indicator. The models are estimated with robust standard errors and results are presented as average marginal effects.

## Results

Table 5 below shows elderly well-being rates by the migration status of adult children. The final column of the table indicates significant differences in the attainment rates between individuals with and without migrant children based on a bivariate means comparison test. The results of analyses comparing well-being rates by age and residency arrangement are available upon request but are excluded here for brevity.

**Table 5:** Well-being Rates by Status of Migrant Child

Indicator	Total	Migrant Child	No Migrant Child	Significance levels
The elderly person is not disabled in terms of basic mobility	59.7 (1.4)	64.4 (2.7)	58.4 (1.6)	*
The elderly person has no difficulties taking medications	74.3 (1.2)	79.3 (2.2)	72.9 (1.4)	**
The elderly person lives in appropriate housing (floor, water, electricity)	74.8 (1.2)	78.0 (2.4)	73.9 (1.4)	
The elderly person has contact with family or friends at least once a week	62.6 (1.3)	67.9 (2.7)	61.2 (1.5)	**
The elderly person is not depressed	59.1 (1.4)	61.4 (2.7)	58.5 (1.6)	
The elderly person has a positive life satisfaction indicator	23.1 (1.1)	25.0 (2.3)	22.5 (1.3)	
Total Multidimensional Index	41.5 (1.4)	47.0 (2.7)	39.9 (1.5)	**

*Source: Authors’ calculations. Standard errors between brackets. Significance levels* * *p* < 0.1, ** *p* < 0.05, *** *p* < 0.01. *Sample weights have been applied to make the data representative for the whole population*

Within the dimension of physical health, approximately 60% of the elderly were able to perform basic functions without difficulty. Not surprisingly, while only 20% of the elderly persons between the ages 60 and 70 had limited mobility, almost 55% of the oldest cohort could not perform basic mobility functions without difficulty. Functional independence, measured by the ability to self-administer medicine, indicated similar differences in attainment according to age. More than 80% of the elderly between 60 and 70 years old attained wellness in this indicator compared to only 65% of those in the oldest cohort. In both indicators, elderly individuals with adult children living abroad achieved significantly higher levels of well-being, particularly in the independence indicator, where over 79.3% of elderly persons with migrant children (compared to 72.9% of those without migrant children) were found to be well.

The domain of housing well-being is comprised of an index measuring the access of the household to appropriate water, electricity, and flooring. Within the total elderly sample, nearly three-quarters could be considered to live in appropriate housing; a slightly greater share of individuals with migrant children had adequate housing, but the difference in outcomes with individuals without migrant children was not statistically significant.

Within the domain of social well-being, an individual was considered to be well if s/he had contact with family or friends at least once a week. Based on this threshold, almost 63% of all elderly persons were considered well. Elderly individuals with migrant children had significantly higher social well-being rates (with 67.9% being well-off) compared to those individuals without migrant children (with 61.2% of such persons being well-off).

The emotional well-being dimension was comprised of indicators of self-reported depression and life satisfaction. Approximately 40% of the total elderly sample reported being depressed, with the oldest cohort experiencing slightly higher depression rates than the total sample. In contrast, well-being rates were extremely low for the life satisfaction indicator: only 23.1% of respondents reported being satisfied with their lives. Differences between age groups were statistically significant, with the youngest cohort experiencing higher well-being rates. There were no significant differences in either depression or life satisfaction based on the migration status of adult children, however.

When well-being rates are calculated for the composite index, only 41.5% of the total elderly population can be identified as multidimensionally well based on the well-being threshold of 70%. A greater share of elderly individuals with an adult migrant child (47%) were considered multidimensionally well than those without a child living abroad (39.9%), and this difference was statistically significant. This finding is robust to different cut-off points. This means that elderly with a migrant child are always better-off regardless the threshold used*.*

Such results provide a preliminary sense of how different aspects of an elderly person’s well-being may differ according to the location of a child, but to account for the other aspects of an individual’s life that may shape well-being, multivariate analysis is needed. Probit models in which the probability of an elderly individual being considered well in each indicator were then estimated. In addition to the migration status of the adult child, other explanatory variables that could explain the indicator well-being outcomes were included in the estimation. These variables included household locale (rural/urban), personal characteristics of the elderly person (sex and age), and household characteristics (such as residency arrangement, highest levels of education in the household, and whether there is an adult child currently living in the household). Table 6 shows the results of the models.^4^

**Table 6:** Multivariate Analysis of Indicator Well-being Rates

	Proper housing	Medication	Mobility	Contact	Not depressed	Satisfied	MWI
Migrant child	0.02	0.05	0.10^**^	0.06	0.03	0.05	0.08^**^
	(0.04)	(0.04)	(0.04)	(0.05)	(0.05)	(0.04)	(0.04)
Child in the HH	-0.02	-0.07^**^	-0.02	-0.03	0.06	0.04	0.01
	(0.03)	(0.03)	(0.03)	(0.04)	(0.04)	(0.03)	(0.03)
No. of children	-0.02^*^	0.00	-0.01	-0.01	-0.02	0.02^**^	-0.03^***^
	(0.01)	(0.01)	(0.01)	(0.01)	(0.01)	(0.01)	(0.01)
Age 70+	-0.03	-0.21^***^	-0.28^***^	-0.17^***^	0.02	-0.04^**^	-0.17^***^
	(0.02)	(0.02)	(0.02)	(0.03)	(0.03)	(0.02)	(0.02)
Male	0.01	0.07^***^	0.13^***^	0.14^***^	0.10^***^	0.02	0.12^***^
	(0.02)	(0.02)	(0.03)	(0.03)	(0.03)	(0.02)	(0.02)
Ethnic	-0.05	-0.09^**^	0.02	0.00	0.09^+^	-0.04	-0.00
Georgian	(0.04)	(0.04)	(0.04)	(0.04)	(0.05)	(0.04)	(0.04)
Urban	0.14^***^	0.10^***^	0.07^***^	0.04	0.05	0.03	0.07^**^
	(0.03)	(0.03)	(0.03)	(0.03)	(0.03)	(0.03)	(0.03)
Migrant	0.06	-0.02	-0.02	-0.02	0.05	-0.06	-0.01
child*urban	(0.06)	(0.05)	(0.06)	(0.06)	(0.06)	(0.05)	(0.05)
Highest Education Level Achieved in the HH (ref. category: lower secondary)							
Post secondary	-0.05	-0.11^**^	0.01	-0.11^*^	-0.09	0.00	-0.02
	(0.05)	(0.05)	(0.05)	(0.06)	(0.06)	(0.06)	(0.05)
Higher	0.08^*^	-0.05	0.09^*^	-0.00	-0.04	0.14^**^	0.06
	(0.04)	(0.05)	(0.05)	(0.05)	(0.06)	(0.05)	(0.05)
Residency Arrangement (ref. category: living alone)							
Living with a partner	0.08^*^	0.10^**^	0.03	-0.02	-0.03	0.12^**^	0.04
	(0.04)	(0.04)	(0.05)	(0.05)	(0.05)	(0.05)	(0.05)
Living with other adults	0.04	0.14^***^	0.12^**^	-0.04	-0.03	0.00	-0.01
	(0.05)	(0.04)	(0.05)	(0.05)	(0.05)	(0.05)	(0.05)
Living in a HH	0.00	0.14^***^	0.13^**^	0.00	0.01	0.05	-0.00
with children	(0.05)	(0.05)	(0.05)	(0.06)	(0.06)	(0.05)	(0.05)
Internally-	-0.04	0.02	-0.04	0.07	-0.24^***^	-0.06	-0.05
displaced HH	(0.05)	(0.05)	(0.06)	(0.06)	(0.06)	(0.05)	(0.05)
Poor HH	-0.11^***^	-0.08^***^	-0.12^***^	-0.14^***^	-0.14^***^	-0.13^***^	-0.38^***^
	(0.02)	(0.03)	(0.03)	(0.03)	(0.03)	(0.03)	(0.02)
HH receives	-0.02	-0.03	-0.03	0.16^***^	0.09^***^	0.06^**^	0.10^***^
remittances	(0.03)	(0.03)	(0.03)	(0.03)	(0.03)	(0.03)	(0.03)
Observations	2202	2202	2202	2202	2202	2202	2202
F stat	9.6	10.9	17.9	12.8	7.1	6.5	30.0
Prob > F	0.00	0.00	0.00	0.00	0.00	0.00	0.00

*Source: Authors’ calculations. dy/dx: marginal effects; standard errors in parentheses; significance levels* *p < 0.1, ** *p* < 0.05, *** *p* < 0.01. *Sample weights have been applied to make the data representative for the whole population*

With the inclusion of other explanatory variables, the migration status of an adult child corresponded to significant differences in the well-being outcomes of elderly individuals in the physical wellbeing dimension and in the overall multidimensional index. In the mobility indicator, elderly individuals with a migrant child had a ten-percentage point higher chance of being considered well than did individuals without migrant children, a result that was significant at the five-percent level. The same applies to the overall multidimensional index, where elderly persons with children living abroad were eight-percentage points more likely to attain well-being. While such differences are not necessarily large, they are notable for being *positive*, which contrasts sharply to the findings of much other research that generally suggested negative relationship between migration and well-being.

Other factors beyond the migration status of adult children also corresponded to meaningful differences in the probability of attaining well-being. Looking first at the different indicators individually, age, sex, residence locale, and poverty status were consistently significant factors. Individuals in the oldest age cohort had much lower probabilities of attaining wellness in the medication, mobility, and contact indicators than did members of the youngest age cohort; this difference was largest in the mobility indicator, where individuals aged 70 or older were predicted to have a 28-percentage point lower chance of being well than those aged 60 to 69. Compared to women, men had higher probabilities of being well in the medication, mobility, contact, and depression indicator by between seven and 14-percentage points. This difference is likely to reflect the much higher proportion of women than men among the oldest individuals. Individuals living in urban (compared to rural) areas had higher probabilities of being well in the housing and physical well-being dimensions, with the greatest difference (of 14-percentage points) occurring in the housing indicator. Those individuals living in households with reported total expenditure below 60% of the sample median (a proxy for income poverty) had much lower probabilities of being well in all indicators, with the greatest differences occurring in the contact and depression indicator. Moreover, receiving remittances increased the likelihood of the elderly being well-off in the contact, depression, and life satisfaction indicators.

As in the analysis of indicator well-being rates, factors such as an individual’s age, sex, area of residence, and poverty status were all contributes to significant differences in achieved multidimensional well-being. Belonging to the youngest cohort (60–70 years old), being male, and living in an urban area were all associated with higher well-being rates. Being in poor household corresponded to a drastically lower probability of being multidimensionally well, with those who lived in low-income^5^ households have a 38-percentage point lower probability of being well than those who lived in higher-income households. In contrast, those individuals who received remittances had a ten-percent point higher probability of being multidimensionally well.

## Discussion

The examination of the relationship between elderly well-being outcomes and the migration of adult children reveals three key trends: 1) there are significant differences in rates of well-being attainment across different domains of well-being, regardless of child migration status; 2) the relationship between migration and well-being varies considerably by domain of well-being being measured, and; 3) migration bears a generally benign relationship to well-being. Taken together, these trends suggest that other factors beyond migration play important roles in shaping well-being outcomes, which can facilitate the design of better interventions that address the unique vulnerabilities and needs of the elderly population.

The analyses revealed that the well-being of the elderly can be decomposed into individual components that signal areas of greater concern based on universally lower rates of attainment, which exist regardless of child migration status. Rates of well-being attainment differed radically by dimension: whereas over 74% of the population aged 60+ passed the threshold for well-ness in the housing dimension, only 23% of the elderly population could be considered well in terms of life satisfaction. These differences reinforce the notion that well-being is comprised of distinct domains that are likely to be effected differently by the ageing process. While the absence of a child may play into that process in specific ways, all members of the population share low well-being rates in some domains, suggesting a role for targeted assistance measures that address particular areas of vulnerability (e.g., poor life satisfaction).

The limited relationship between the absence of an adult child through migration and the well-being outcomes of their elderly parents is somewhat surprising given the results of much past research, which largely suggests deterioration of wellness following migration. A child’s migration was never associated with worse well-being outcomes; on the contrary, the analyses suggested that elderly individuals with a migrant child had higher probabilities of attaining well-ness in the physical well-being domain as well as in the overall multidimensional index. The most striking association between migration and well-being outcomes was in the domain of mobility, where elderly persons with an adult migrant child had a ten-percentage-point higher probability of being well.

The overall benign association between migration and elderly well-being suggests that the mere presence or absence of a child through migration is not necessarily the most imperative factor that shapes well-being outcomes. Factors such as being in the oldest age cohort, being female, living in a rural area, and living in a poor household were all strongly, negatively associated with the attainment of well-being across almost all domains. Compared to child migration, which bore only positive associations with certain domains of well-being and of relatively low magnitude, these factors were associated with much larger differences in the probability of individuals being well. These factors can again suggest sources of vulnerability that can be addressed through better-targeted support measures.

These results suggest that the context in which an elderly person ages matters for the attainment of well-being, and the role of migrants within families and as providers of both financial and non-financial care resources is especially important to understand in this regard. In Georgia, it is common for elderly individuals to live with their adult children, particularly when they need physical or financial assistance. Such elder-care norms will likely encourage siblings (and other members of the extended family) to coordinate migration and care decisions to ensure that elderly persons are not left without assistance, a negotiation process documented in other countries such as Moldova (Stöhr 2013). Studies conducted in other contexts, such as Taiwan and China, have suggested that strong intergenerational support and filial care norms can ensure that elderly persons receive emotional, instrument, and financial support from close relatives even when the family context changes though factors such as migration (Lin and Yi 2011). Such processes are likely also at play in Georgia, which may explain why the migration of a child is not associated with worse well-being outcomes.

An additional explanation for this trend, however, relates to self-selection and reserve causality. Migrants are not randomly chosen from the population; migrants are likely to have characteristics that distinguish them from other members of the population. This can include having healthy parents or supportive siblings. Individuals with parents who have low levels of well-being and who do not have siblings that are able to provide care to their parents may be unlikely to enter migration in the first place. Higher rates of mobility among the elderly with migrant children, as an example, may therefore reflect pre-migration health status rather than any benefit created by having a child abroad. Migration may also support active ageing processes, however; elderly persons who assume greater responsibilities relating to child- or home-care following the migration of an adult child may engage in more physical activity, which has been strongly correlated with the maintenance of functional capacity into older age. Migrants may also provide advice or health-related information (Taylor 1999) as well as financial remittances that can help their elderly kin better navigate the challenges associated with ageing (through, e.g., purchase of anti-inflammatory medications).

This discussion highlights two limitations of the analytical method deployed in this analysis. First, endogeneity could not be controlled for in this analysis, which implies only that correlation rather than causation can be inferred. Second, the elderly population was divided according to whether or not they had any child in migration, which did not account for the presence and location of other children. This analysis can therefore be extended by better mapping the family situations in which an elderly person lives, particularly by identifying the distance between an elderly individuals and his/her adult children.

## Appendix

**Table 7:** Descriptive statistics of variables used in the analysis

Variable	Mean	SD	Min	Max
Age 60–69	0.46	0.50	0	1
Male	0.36	0.48	0	1
Urban	0.53	0.50	0	1
Ethnic Georgian	0.89	0.30	0	1
Lower secondary	0.06	0.23	0	1
Post-secondary education	0.17	0.38	0	1
Higher education	0.77	0.42	0	1
Living alone	0.08	0.27	0	1
Living with a partner	0.10	0.30	0	1
Living with other adults	0.46	0.50	0	1
Living in a HH with children	0.36	0.48	0	1
Internally-displaced HH	0.06	0.23	0	1
Poor HH	0.36	0.48	0	1
HH receives remittances	0.26	0.44	0	1
Number of children	2.3	1.14	0	11
Child in the HH	0.55	0.50	0	1
Proper housing	0.76	0.43	0	1
Medication	0.75	0.43	0	1
Mobility	0.62	0.49	0	1
Contact	0.69	0.46	0	1
Not depressed	0.62	0.49	0	1
Satisfied	0.26	0.44	0	1
MWI	0.48	0.50	0	1

**Table 8:** Factor analysis statistics

Factor loadings and unique variances		
Variable	Factor 1	Uniqueness
Dress without help	0.8528	0.2727
Go to the bathroom without help	0.9144	0.1638
To bathe	0.9115	0.1692
To get out of bed	0.8910	0.2061
To walk across the room	0.8109	0.3425
To stand up from sitting position in a chair without help	0.8052	0.3517
Scoring coefficients		
Variable	Factor 1	
Dress without help	0.13430	
Go to the bathroom without help	0.25364	
To bathe	0.23474	
To get out of bed	0.21218	
To walk across the room	0.13639	
To stand up from sitting position in a chair without help	0.11491	

## Footnotes

1 With the exception of the semi-autonomous regions of Abkhazia and South Ossetia.

2 For more information, see: http://www.gallup.com/poll/122453/understanding-gallup-uses-cantril-scale.aspx

3 As we are interested in the analysis of individual domains, we prefer the counting approach to other methods such as principal component analysis

4 Descriptive statistics on the variables used in the regressions are shown in the appendix

5 We use expenditure as a proxy for income as income in this survey is under-represented and expenditure constitutes a better measure of economic wellbeing.
